# AI in Fracture Detection: A Cross-Disciplinary Analysis of Physician Acceptance Using the UTAUT Model

**DOI:** 10.3390/diagnostics15162117

**Published:** 2025-08-21

**Authors:** Martin Breitwieser, Stephan Zirknitzer, Karolina Poslusny, Thomas Freude, Julia Scholsching, Karl Bodenschatz, Anton Wagner, Klaus Hergan, Matthias Schaffert, Roman Metzger, Patrick Marko

**Affiliations:** 1 Department for Orthopedic Surgery and Traumatology, Paracelsus Medical University, 5020 Salzburg, Austria; 2 Department for Pediatric Surgery, Paracelsus Medical University, 90471 Nürnberg, Germany; 3 Department for Radiology, Paracelsus Medical University, 5020 Salzburg, Austria; 4 Department for Pediatric Surgery, Paracelsus Medical University, 5020 Salzburg, Austria

**Keywords:** artificial intelligence, AI, fracture detection, UTAUT, survey, physicians, acceptance, clinical decision support system, CDSS, emergency care, diagnostic tools

## Abstract

**Background/Objectives**: Artificial intelligence (AI) tools for fracture detection in radiographs are increasingly approved for clinical use but remain underutilized. Understanding physician attitudes before implementation is essential for successful integration into emergency care workflows. This study investigates the acceptance of an AI-based fracture detection tool among physicians in emergency care settings, using the Unified Theory of Acceptance and Use of Technology (UTAUT) model. **Methods**: A cross-sectional, pre-implementation survey was conducted among 92 physicians across three hospitals participating in the SMART Fracture Trial (ClinicalTrials.gov: NCT06754137). The questionnaire assessed the four core UTAUT constructs—performance expectancy (PE), effort expectancy (EE), social influence (SI), facilitating conditions (FC)—and additional constructs such as attitude toward technology (AT), diagnostic confidence (DC), and workflow efficiency (WE). Responses were collected on a five-point Likert scale. Structural equation modeling (SEM) and confirmatory factor analysis (CFA) were performed to assess predictors of behavioral intention (BI). **Results**: PE was the strongest predictor of BI (β = 0.5882, *p* < 0.001), followed by SI (β = 0.391, *p* < 0.001), FC (β = 0.263, *p* < 0.001), and EE (β = 0.202, *p* = 0.001). These constructs explained a substantial proportion of variance in BI. WE received the lowest ratings, while internal consistency for SI and BI was weak. Moderator analyses showed prior AI experience improved EE, whereas more experienced physicians were more skeptical regarding WE and DC. However, none of the moderators significantly influenced BI. **Conclusions**: Physicians’ intention to use AI fracture detection is primarily influenced by perceived usefulness and ease of use. Implementation strategies should focus on intuitive design, targeted training, and clear communication of clinical benefits. Further research should evaluate post-implementation usage and user satisfaction.

## 1. Introduction

In recent years, artificial intelligence (AI) has become increasingly embedded in everyday life and has assumed a central role in both scientific research and real-world applications. In medicine, AI-based technologies are being integrated into clinical workflows with the aim of improving diagnostic accuracy, optimizing treatment planning, and enhancing the efficiency of healthcare delivery [1].

Radiology, in particular, has emerged as a leading domain for AI implementation due to its reliance on high-volume data interpretation [2,3]. AI algorithms, trained on large-scale datasets, are now capable of detecting pathological findings in medical images, recognizing rare conditions, and offering evidence-based treatment suggestions in real time [4,5,6]. The potential of AI to reduce diagnostic errors—estimated to affect 5–15% of all medical decisions—and to increase clinical throughput underscores its transformative potential across both inpatient and outpatient settings [7,8].

Among clinical environments, emergency departments (EDs) are especially well-suited for AI-supported systems due to their high patient turnover, limited availability of specialists, and demand for rapid and accurate decision-making [9]. One key area of vulnerability is fracture detection on conventional radiographs, where diagnostic oversight remains a significant concern. Studies report miss rates ranging from 2% to 9%, highlighting the clinical importance of improving detection accuracy [7].

In response to these challenges, several AI-powered diagnostic tools have received regulatory approval for acute fracture detection in recent years, including the Aidoc Fracture Detection Module (Tel Aviv, Israel) and BoneView by Gleamer (Saint-Mandé, France). These tools, designed as clinical decision support systems (CDSS), employ convolutional neural network (CNN) architectures to autonomously analyze radiographic images and flag critical findings—such as vertebral fractures, pulmonary embolisms, or intracranial hemorrhages—in real time [10]. Despite regulatory clearance and promising performance metrics, the widespread adoption of such AI tools remains limited. Reported barriers include challenges with system integration into hospital infrastructure, non-intuitive user interfaces, insufficient training, and concerns about automation bias or overreliance on AI outputs [11,12].

In addition to technical and infrastructural challenges, clinician acceptance has emerged as a pivotal determinant for the successful implementation of AI-based tools in clinical settings. Factors such as perceived reliability, clinical utility, and compatibility with existing workflows significantly influence adoption decisions [13]. Within the European Union, the significance of these human factors is further emphasized by the AI Act and Medical Device Regulation (MDR), which mandate transparency, user training, and accountability as prerequisites for safe and ethical AI deployment in healthcare [2,14].

To systematically examine and predict technology adoption behavior, several theoretical frameworks have been developed. Among the most influential is the Unified Theory of Acceptance and Use of Technology (UTAUT), introduced by Venkatesh et al. in 2003 [15]. Synthesizing eight prior models, UTAUT identifies four key predictors of behavioral intention to use technology: performance expectancy (PE), effort expectancy (EE), social influence (SI), and facilitating conditions (FC). These predictors are further moderated by demographic and experiential variables such as age, gender, professional experience, and voluntariness of use. The UTAUT framework has been extensively validated in healthcare research, including studies on clinician and patient adoption of digital innovations [16,17,18]. Applications range from telemedicine platforms [19] to digital learning environments [20]. UTAUT’s adaptability has also allowed for extensions and modifications, such as age-specific analyses by Palas et al. [21], and patient-focused perspectives explored by Zhang et al. [22].

Despite the recognized promise of AI in radiologic diagnostics, particularly for fracture detection, empirical studies exploring clinician acceptance prior to clinical implementation remain scarce. Existing research often suffers from small sample sizes, technological heterogeneity, and insufficient theoretical grounding. Moreover, little is known about how acceptance varies by clinical specialty, years of experience, or previous exposure to AI technologies [23,24,25]. Understanding these variables—and how they influence behavioral intention (BI) and subsequent use behavior (UB)—is critical for designing targeted implementation strategies and achieving successful integration into clinical practice.

To address these gaps, the present study conducts a pre-implementation assessment of physicians’ acceptance of AI-assisted fracture detection as part of the SMART Fracture Trial, a multicenter randomized controlled trial conducted in emergency care settings (ClinicalTrials.gov Identifier: NCT06754137). As one of the first theory-driven investigations in this area, the study aims to generate actionable insights to inform clinician training, facilitate effective implementation, and support the broader integration of AI tools into routine clinical workflows.

## 2. Materials and Methods

### 2.1. Study Design and Setting

This multicenter, cross-sectional survey represents the baseline (pre-implementation) assessment of physician attitudes within the SMART Fracture Trial—a prospective, randomized controlled study examining the influence of AI-assisted fracture-detection software on emergency department workflows. Data were collected from three sites: the Departments of Orthopedics and Traumatology, Pediatric Surgery, and Radiology at the University Hospital Salzburg (Austria); the corresponding departments at the University Hospital Nuremberg (Germany); and the Department of Orthopedics and Traumatology at the Regional Hospital Hallein (Austria). The two university hospitals are Level I trauma centers, each managing approximately 180,000 emergency presentations and more than 120,000 radiographic examinations annually, while Hallein functions as a regional acute-care hospital with approximately 50,000 emergency presentations and over 30,000 radiographic examinations per year. As these three sites are located within one Alpine healthcare region, the findings reflect a geographically confined physician cohort.

### 2.2. Survey Design and Conceptual Framework

**Figure 1:**
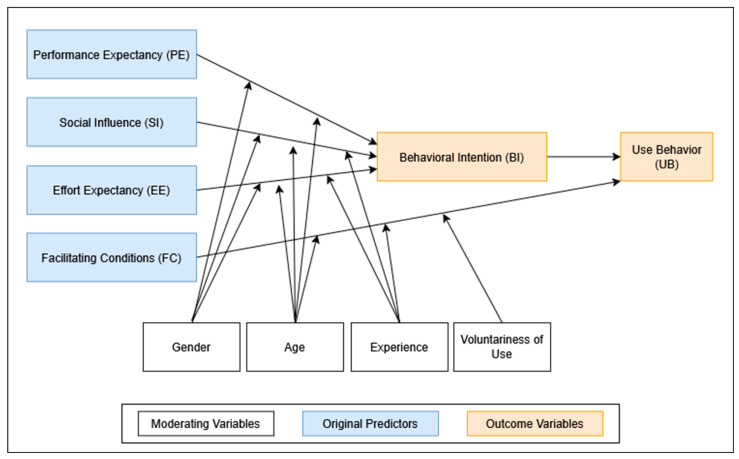
Unified Theory of Acceptance and Use of Technology (UTAUT) framework adapted from Venkatesh et al., 2003 [15].

The survey instrument was based on the Unified Theory of Acceptance and Use of Technology (UTAUT), proposed by Venkatesh et al. (2003) [15] (see Figure 1). The survey was administered to physicians participating in the SMART Fracture Trial (*n* = 92) and included 21 items covering the five canonical UTAUT constructs: Performance Expectancy (PE), Effort Expectancy (EE), Social Influence (SI), Facilitating Conditions (FC), and Behavioral Intention (BI). Responses were recorded on a five-point Likert scale (1 = “strongly disagree”; 5 = “strongly agree”).

**Figure 2:**
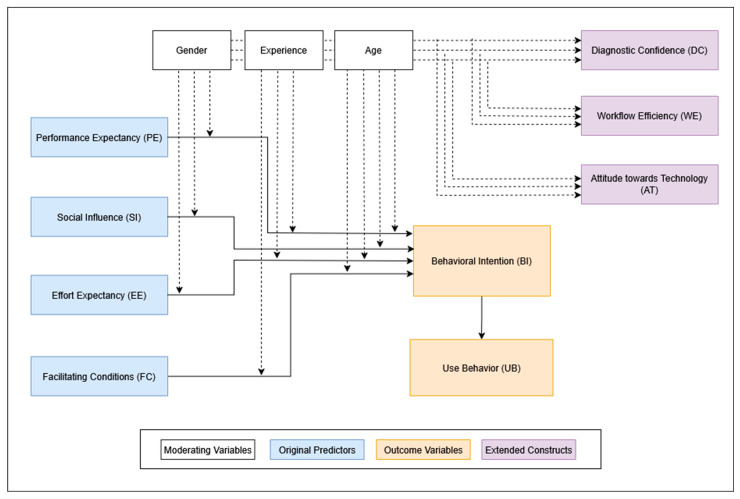
Adapted UTAUT framework used in this study.

In line with the original UTAUT framework, these five constructs were modeled as predictors of BI, which was assumed to influence actual Use Behavior. To adapt the UTAUT model for the clinical context of AI-assisted fracture detection, we added three additional constructs: Attitude toward Technology (AT), Workflow Efficiency (WE), and Diagnostic Confidence (DC) (see Figure 2). These were chosen to capture clinically relevant dimensions such as openness to innovation, perceived impact on daily workflow, and confidence in diagnostic support. To keep the statistical model predicting BI reasonably simple, AT, WE, and DC were not directly included as predictors but were analyzed in relation to various moderating variables.

The questionnaire also captured demographic data including age and gender, as well as clinical specialty, professional grade, years of postgraduate experience, and prior experience with AI in general and AI-based fracture detection in particular. These variables are well-established moderators within the UTAUT framework. Building on this foundation, we incorporated additional constructs relevant to AI in clinical settings, as recommended by Venkatesh, who emphasizes the importance of adapting UTAUT to the contextual, technological, and psychological characteristics of the target application [26].

The construct Voluntariness of Use was deliberately excluded. As the survey was conducted before the AI tool was implemented, no usage policy had been established, and participants evaluated the tool hypothetically. Moreover, empirical evidence suggests that voluntariness has limited predictive value in protocol-driven clinical environments, such as hospitals, where usage decisions are often determined by institutional policies rather than personal discretion [27].

The original English items were translated into German using OpenAI’s large language model (ChatGPT) and subsequently reviewed by a language editor with statistical expertise to ensure semantic and conceptual equivalence. Face validity was established through pilot testing with five physicians not involved in the main study, resulting in only minor wording adjustments. The final German questionnaire is included in the project files.

### 2.3. Participant Recruitment and Data Handling

All licensed physicians actively involved in fracture diagnosis or management within the participating departments during the SMART Trial recruitment period were eligible to participate. Exclusion criteria included medical students, visiting observers, and physicians not directly involved in patient care. From 1 March to 1 June 2025, each eligible clinician was invited to complete the survey. The invitation outlined study objectives and instructions. Respondents were asked to generate a four-character linkage code to enable anonymous pairing with the post-trial survey. Non-responders received reminders on days 14 and 28. Participation was voluntary, and no incentives were offered.

Paper-based questionnaires were double-entered by trained research staff. All records were anonymized and stored on EU-based servers in full compliance with the General Data Protection Regulation (GDPR). Only de-identified data were used for analysis.

### 2.4. Outcomes

The primary outcome was physicians’ Behavioral Intention (BI) to use AI-assisted fracture-detection software. We examined the extent to which BI could be predicted by the four UTAUT constructs—PE, EE, SI, and FC—and report the percentage of variance in BI explained by these predictors.

As a secondary aim, we evaluated whether the effects of PE, EE, SI, and FC on BI varied across four background variables: clinical specialty (radiology vs. surgical specialties), professional experience (<7 years vs. ≥7 years), prior experience with AI in general, and prior experience with AI-assisted fracture detection. If these variables significantly influenced the strength of the relationships, they were considered important moderators.

### 2.5. Statistical Analysis

The final analytic dataset included 92 physicians—sufficient for structural modeling under the guideline of approximately 10 observations per estimated parameter [28]. To ensure model stability, we trimmed the structural equation model (SEM) and treated subsequent results as exploratory.

All analyses were conducted in R version 4.4.1 using the *lavaan*, *semTools*, *psych*, *likert*, and *ggplot2* packages. The analytic workflow (see Figure 1) followed five sequential steps:

1. Descriptive summaries and scale reliability (Cronbach’s α; Spearman–Brown for two-item scales);
2. Confirmatory factor analysis (CFA) using robust diagonally weighted least squares (DWLS; ordered = TRUE, std.lv = TRUE), appropriate for ordinal data and small samples (<100 cases) [29];
3. Trimmed SEM: BI_mean ≈ PE + EE + FC + SI;
4. Ordinary least squares regression analyses testing three binary moderators (sex, >6 years of experience, prior AI-fracture exposure);
5. Cross-study comparison of path coefficients (Appendix A, Table A1).

Simplification strategies included the following: (i) no moderators within the SEM, (ii) dichotomization of moderator variables, and (iii) replacing the latent BI factor with a composite score (BI_mean), calculated as the arithmetic mean of its two items. Each latent construct comprised only two or three indicators to maintain a favorable parameter-to-sample ratio.

Descriptive and subgroup analyses followed standard procedures. Pairwise deletion was used for <1% missing values, and listwise deletion removed two incomplete responses from model estimation. Measurement quality was assessed via Cronbach’s α (target ≥ 0.70) and CFA fit indices (CFI ≥ 0.95, TLI ≥ 0.95, RMSEA ≤ 0.06, SRMR ≤ 0.08).

All statistical tests were two-sided, with significance set at *p* < 0.05. Factor loadings were classified as strong (≥0.70), moderate (≥0.50), or weak (<0.50).

### 2.6. Ethical Considerations

The SMART Trial was approved by the Ethics Committee of the Federal State of Salzburg (EK No. 1135/2024) on 8 January 2025. Participation in the pre-trial survey was voluntary; completion and return of the questionnaire were taken as informed consent. No identifiable personal data were collected, and the study conformed to the principles of the Declaration of Helsinki (2013 revision).

### 2.7. Use of Generative AI

OpenAI’s ChatGPT-4o (San Francisco, CA, USA), DeepSeek (Shanghai, China), and Grammarly AI (San Francisco, CA, USA) were used to translate and adapt the UTAUT questionnaire into German and to assist with grammar and style editing of the manuscript. These tools were not involved in study design, data collection, statistical analysis, or interpretation.

### 2.8. Transparency, Reproducibility, and Data Availability

All anonymized data, the German and English survey instruments, and the full R analysis script will be made available upon reasonable request, in line with MDPI’s open data policy.

## 3. Results

### 3.1. Response Rate and Participant Characteristics

A total of 92 physicians completed the baseline questionnaire, yielding a response rate of 83.8% among those invited during the SMART Trial pre-implementation phase. The mean age of respondents was 41.1 years (SD = 11.3), with a median age of 38.0 years.

Of the respondents, 58 (63.0%) identified as male and 34 (37.0%) as female. Clinical specialties were distributed as follows: 27 (29.3%) in Pediatric Surgery, 51 (55.4%) in Orthopedics or Trauma Surgery, 12 (13.0%) in Radiology, and 2 (2.2%) in other disciplines. Regarding professional roles, 44 (47.8%) were board-certified consultants, 41 (44.6%) were residents or physicians in training, and 4 (4.3%) worked as hospital-based general practitioners. As males and surgical specialists predominated (63% and 85%, respectively), the dataset is demographically unbalanced.

In terms of clinical experience, 43 (46.7%) reported more than 10 years of postgraduate experience, 7 (7.6%) had 7–10 years, 33 (35.9%) had 1–6 years, and 9 (9.8%) had less than 1 year. Regarding prior exposure to artificial intelligence in clinical practice, 27 (29.3%) reported no experience, 44 (47.8%) limited exposure, 13 (14.1%) moderate exposure, and 8 (8.7%) extensive exposure. More specifically, 51 physicians (55.4%) had no prior experience with AI-based fracture detection software such as Aidoc’s CE-marked fracture detection module (Version 1.x), while 27 (29.3%) reported limited, 10 (10.9%) moderate, and 4 (4.3%) extensive experience (see Table 1).

### 3.2. Construct Scores and Internal Consistency

**Figure 3:**
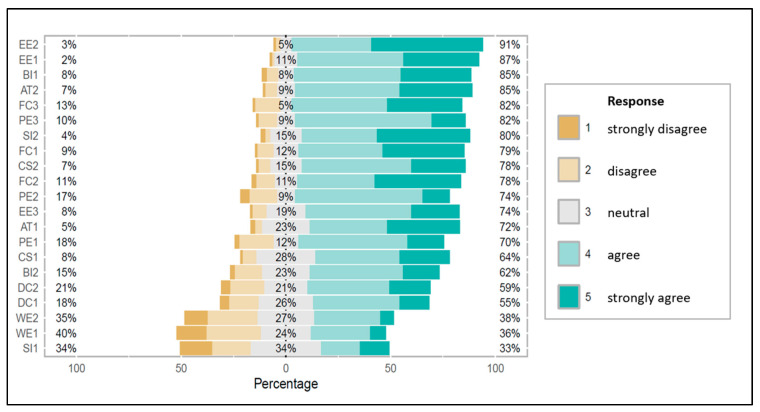
Frequency distribution of participant ratings across UTAUT and extension items on a 5-point Likert scale.

All 21 UTAUT items were assessed using five-point Likert scales (see Figure 3). Mean scores ranged from 2.94 (WE) to 4.16 (EE), with standard deviations between 0.63 and 1.06, reflecting moderate variability in response patterns. The constructs with the highest mean agreement were EE (mean = 4.16, SD = 0.63), AT (mean = 4.05, SD = 0.80), and PE (mean = 3.71, SD = 0.83). The lowest agreement was observed for WE (mean = 2.94, SD = 1.05), indicating greater skepticism about the software’s potential impact on throughput and time savings (see Table 2).

Internal consistency was assessed using Cronbach’s α for each of the nine latent constructs. The highest reliability was observed for CS (α = 0.854) and WE (α = 0.780), both interpreted as good. PE (α = 0.794) and DC (α = 0.783) also demonstrated good reliability. EE (α = 0.686), FC (α = 0.654), and AT (α = 0.695) showed borderline acceptable reliability, suggesting moderate item consistency with potential for refinement in future studies.

In contrast, the two-item scales SI (α = 0.537) and BI (α = 0.560) fell below the conventional acceptability threshold, indicating poor internal consistency. This is not uncommon for two-item scales; notably, all CFA loadings exceeded 0.62 and overall model fit remained acceptable, indicating that content validity was not compromised (see Section 3.3).

### 3.3. Confirmatory Factor Analysis

A confirmatory factor analysis (CFA) was conducted to evaluate the construct validity of the variables derived from the UTAUT model (see Table 3).

Three of the four fit indices—CFI (0.993), TLI (0.990), and SRMR (0.080)—met or were very close to commonly accepted thresholds for good model fit. The RMSEA (0.068) slightly exceeded the recommended threshold (≤0.06), indicating a marginal degree of model misfit.

All latent constructs showed statistically significant factor loadings (*p* < 0.001), indicating strong relationships between observed indicators and their respective latent dimensions. Overall, the hypothesized factor structure provided a reasonably good representation of the observed data, supporting its use for further model-based analyses, albeit with caution regarding residual error.

### 3.4. Structural Equation Modeling

To test the core assumptions of the UTAUT model, an SEM was specified with PE, EE, FC, and SI modeled as direct predictors of Behavioral Intention (BI). Due to convergence issues related to sample size and weak internal consistency of some constructs, BI could not be estimated as a latent variable. Instead, the mean of its two items (BI_mean) was used as a single observed outcome. This allowed estimation of a simplified SEM using the DWLS estimator.

All four canonical UTAUT predictors were statistically significant predictors of BI_mean. PE was the strongest predictor (β = 0.588, *p* < 0.001), followed by SI (β = 0.391, *p* < 0.001), FC (β = 0.263, *p* < 0.001), and EE (β = 0.202, *p* = 0.001) (Table 4).

These results provide strong support for the UTAUT model in explaining physicians’ intention to use AI-assisted fracture detection tools in a clinical setting.

### 3.5. Moderator Effects

To explore whether physician characteristics influence the latent UTAUT constructs and the outcome variable (BI_mean), moderator analyses were performed using separate regression models using three binary moderators: gender, clinical experience (≤6 vs. >6 years), and prior experience with AI-based fracture detection. The results are presented below for each moderator.

#### 3.5.1. Gender

None of the gender-based effects reached statistical significance at *p* < 0.05, although associations with Clinical Satisfaction (*p* = 0.075) and Diagnostic Confidence (*p* = 0.109) approached significance (Appendix A Table A1). This suggests a possible trend toward higher satisfaction and confidence among male physicians, though the effects were not robust. Gender had no effect on BI_mean.

#### 3.5.2. Years of Experience

Physicians with more than six years of experience reported significantly higher perceived facilitating conditions (*p* = 0.038) but lower workflow efficiency (*p* = 0.030) and lower diagnostic confidence (*p* = 0.023) (Appendix A Table A2). These findings suggest that more experienced clinicians recognize stronger institutional support but remain more skeptical about AI’s efficiency and its ability to enhance diagnostic certainty. No significant effect was observed on BI_mean.

#### 3.5.3. Experience with AI-Based Fracture Detection

The only significant moderation effect of prior AI-based fracture detection experience was on Effort Expectancy (*p* = 0.008), indicating that physicians with hands-on experience perceived the software as easier to use (Appendix A Table A3). No other latent constructs or BI_mean were significantly affected, though most coefficients trended positive.

## 4. Discussion

This multicenter, cross-sectional study assessed physicians’ acceptance of an AI-assisted fracture detection tool prior to its clinical deployment, applying an extended UTAUT model. A total of 92 physicians from orthopedics and trauma surgery, pediatric surgery, and radiology completed the pre-implementation survey as part of the SMART Fracture Trial, a randomized controlled study evaluating the impact of AI-supported fracture detection in emergency care.

The findings strongly support the UTAUT framework: All four core constructs—Performance Expectancy (PE), Effort Expectancy (EE), Social Influence (SI), and Facilitating Conditions (FC)—were significant predictors of Behavioral Intention (BI). These results align with prior UTAUT applications in clinical AI, including decision-support tools, where PE and EE have consistently emerged as the strongest determinants of BI and key drivers of technology adoption [30,31,32,33,34].

Among the predictors, PE exerted the greatest influence on BI, indicating that physicians are more likely to adopt AI tools when they anticipate clear improvements in clinical performance. This mirrors earlier findings in radiology, primary care, and AI-enabled decision-making, where PE was consistently identified as the most important factor shaping intention to use digital health technologies [30,31,32,33]. In the present study, PE also ranked among the highest-scoring constructs, underscoring its central role in driving adoption.

EE was another strong predictor of BI and received high agreement scores. Previous UTAUT-based studies in clinical AI have emphasized that ease of use is essential for fostering trust and encouraging adoption, especially when technology demonstrably reduces cognitive workload [31,32,33,35,36]. Moderator analysis revealed that physicians with prior experience in AI-assisted fracture detection reported significantly higher EE, suggesting that direct exposure enhances perceived usability.

Although FC had a smaller effect size than PE or EE, it remained a meaningful predictor of BI. This is consistent with evidence that institutional readiness, infrastructure, and IT support facilitate the transition from intention to actual use [33]. Physicians with more than six years of experience rated FC significantly higher, possibly reflecting a greater awareness of available institutional resources to support AI implementation.

SI also significantly predicted BI, but its internal consistency was low. This finding is consistent with Lee et al. (2023), who observed similarly limited effects of social influence on AI acceptance among emergency physicians [37]. In fast-paced, high-pressure environments such as emergency care, clinical decision-making tends to be autonomous, potentially reducing the influence of peer or supervisory expectations [38,39].

The BI construct itself showed relatively low internal consistency, suggesting that the two survey items used may not have fully captured a single underlying dimension. This limitation likely reflects the pre-implementation context, where physicians were asked to assess their intentions without hands-on experience with the tool. Similar challenges have been reported in other pre-implementation studies, where BI is shaped more by assumptions or external messaging than by direct experience [32]. Despite this, BI scores were relatively high, indicating general openness to the technology. Moderator analyses revealed no significant effects of gender, professional experience, or prior exposure to AI-based fracture detection on BI. A comparative overview of our findings with those of other AI studies is provided in Appendix A Table A4.

Beyond the core UTAUT constructs, this study examined three additional variables—Attitude toward Technology (AT), Workflow Efficiency (WE), and Diagnostic Confidence (DC)—to provide a more nuanced understanding of clinicians’ expectations before implementation. Although not part of the original UTAUT model, these constructs hold substantial clinical relevance. A positive AT can facilitate openness and reduce resistance to new digital systems. WE captures the extent to which AI is expected to streamline clinical routines—an especially critical factor in emergency settings. DC reflects confidence in clinical judgment when aided by AI, a determinant of both decision-making and patient safety [35].

Moderator analysis revealed that physicians with more than six years of experience, while reporting higher FC, gave lower ratings for both DC and WE. This contrast may reflect experience-driven skepticism: less experienced clinicians may be more receptive to new technologies and more inclined to rely on AI support, whereas more experienced clinicians may be more aware of potential limitations, risks, and the possibility of automation bias. Such attitudes are consistent with prior research showing that clinical experience, perceived risk, and prior exposure shape how decision-support tools are evaluated [40,41,42].

WE received the lowest agreement scores of all constructs. Although AI tools are often promoted as improving efficiency and reducing workload, many participants were uncertain whether these benefits would materialize in daily practice. This caution mirrors findings from emergency care studies, where clinicians tend to reserve judgment on workflow impact until a technology has demonstrated value in routine use [33].

In contrast, AT was rated highly, suggesting a generally positive stance toward technological innovation among respondents. Combined with the strong ratings for PE and EE, these results indicate that acceptance is greatest when AI tools are perceived as clinically valuable, user-friendly, and supported by the broader institutional framework.

These findings underscore that effective implementation strategies must extend beyond leadership endorsement or regulatory approval. Successful adoption will require targeted peer-led training, opportunities for hands-on use, and transparent communication about the system’s capabilities and limitations. A gradual rollout, coupled with continuous feedback, may help align expectations with actual performance and increase acceptance through direct demonstration in clinical practice.

Several limitations must be acknowledged. First, the study exclusively reflects physicians’ perspectives and does not capture patient views, which are also relevant to the adoption of AI in clinical decision-making. Issues such as patient trust in AI-generated diagnoses, disclosure of AI involvement, and perceived transparency remain unexplored. Second, the cross-sectional design captures only stated intentions, not actual usage behavior. Many respondents had no direct experience with the AI tool, which may have affected their ratings, particularly for constructs such as BI. Consequently, no conclusions can be drawn about whether initial intentions will translate into sustained use after implementation.

Third, generalizability is limited by the specific clinical and technological context of this study. The focus on AI-assisted fracture detection in emergency care means results may not extend to other specialties, clinical settings, or AI applications, where determinants of acceptance may differ. Future research should assess whether the identified patterns hold in other domains, especially where time constraints and diagnostic complexity vary.

Fourth, despite careful translation of the UTAUT items into German, subtle differences in meaning or cultural interpretation may have influenced responses, particularly for constructs such as SI and FC. Constructs with only two items were especially susceptible to reliability issues. In addition, voluntary participation may have introduced selection bias toward respondents more favorably inclined toward digital technologies.

Future research should replicate these findings in larger and more diverse samples to enhance generalizability and allow more detailed subgroup analyses—particularly for constructs with lower reliability such as SI and BI. The planned second phase of this study will evaluate actual usage patterns following implementation, including usage frequency, clinical contexts, and user profiles, as well as follow-up survey data on satisfaction, perceived impact on diagnostic workflow, and changes in diagnostic confidence. This post-implementation phase will help determine whether initial acceptance translates into real-world utilization and sustained value and will identify barriers and opportunities for improving training, integration, and long-term adoption.

## 5. Conclusions

This study explored how physicians perceive and accept the use of AI-based tools for fracture detection before such systems are actually introduced into clinical practice. The results show that most physicians view these tools positively, especially when they believe the system will help them make better clinical decisions (PE—strongest predictor of BI) and is easy to use (EE—also significantly associated with BI). Confidence in the usefulness and user-friendliness of the tool emerged as the most important factors influencing whether doctors would consider using it in the future.

We acknowledge that the modest sample size limits precision; hence, all numeric estimates should be viewed as exploratory, yet the observed trends align with prior domain knowledge and revealed areas of uncertainty. Many doctors were unsure whether the tool would truly save time or improve their workflow (WE—lowest rated construct). In addition, the opinions of colleagues and superiors—often considered influential—played a smaller role than expected (SI—significant path to BI, but low internal consistency). These insights suggest that simply having a good product is not enough; successful implementation depends on clear communication, realistic expectations, and hands-on training that fits into everyday routines.

To improve acceptance and future use, hospitals and developers should focus on three key actions: (1) highlight how the tool improves accuracy and supports decision-making (PE), (2) ensure the system is intuitive and easy to use (EE), and (3) provide real-world opportunities for physicians to test and integrate the tool into their work (FC—moderate influence on BI). Follow-up studies after implementation will be crucial to understand how these early expectations translate into actual behavior and satisfaction once the AI system becomes part of clinical practice.

## Figures and Tables

**Table 1:** Participant characteristics.

Variable	Category	*n*	%
Gender	Female	34	37.0%
	Male	58	63.0%
Specialty	Orthopedics/Trauma	51	55.4%
	Radiology	12	13.0%
	Pediatric Surgery	27	29.3%
	General Practitioners	2	2.2%
Professional grade	Consultant	44	47.8%
	Resident/Registrar	41	44.6%
	Other	7	7.6%
Years of experience	<1 year	9	9.8%
	1–6 years	33	35.9%
	7–10 years	7	7.6%
	≥10 years	43	46.7%
Prior AI exposure	None	27	29.3%
	Limited	44	47.8%
	Moderate	13	14.1%
	Extensive	8	8.7%
Experience with AI Fracture detection	None	51	55.4%
	Limited	27	29.3%
	Moderate	10	10.9%
	Extensive	4	4.3%

**Table 2:** Mean agreement scores and internal consistency (Cronbach’s α) by construct.

Construct	Items	Internal Consistency (Cronbach’s α)	Mean	Standard Deviation	Interpretation
Performance Expectancy (PE)	3	0.794	3.71	0.83	Good
Effort Expectancy (EE)	3	0.686	4.16	0.63	Acceptable
Social Influence (SI)	2	0.537	3.58	0.91	Poor
Facilitating Conditions (FC)	3	0.654	4.06	0.77	Acceptable
Attitude towards Technology (AT)	2	0.695	4.05	0.80	Acceptable
Behavioral Intention (BI)	2	0.560	3.85	0.79	Poor
Diagnostic Confidence (DC)	2	0.783	3.50	0.98	Acceptable
Workflow Efficiency (WE)	2	0.780	2.94	1.05	Good
Clinical Satisfaction (CS)	2	0.854	3.73	0.84	Good

**Table 3:** CFA fit indices with thresholds.

Fit Index	Value	Threshold
Comparative Fit Index (CFI)	0.993	≥0.95
Tucker–Lewis Index (TLI)	0.990	≥0.95
Root Mean Square Error of Approximation (RMSEA)	0.068	≤0.06
Standardized Root Mean Square Residual (SRMR)	0.08	≤0.08

**Table 4:** Regression coefficients predicting BI_mean from canonical UTAUT predictors.

Predictor	Estimate β	SE	Z	*p*-Value
Performance Expectancy (PE)	0.5882	0.069	8.495	<0.001
Effort Expectancy (EE)	0.2015	0.061	3.298	0.001
Facilitating Conditions (FC)	0.2632	0.070	3.773	<0.001
Social Influence (SI)	0.3906	0.089	4.404	<0.001

## Data Availability

The data presented in this study are available upon request from the corresponding author.

## Abbreviations

The following abbreviations are used in this manuscript:

AI	Artificial Intelligence
AT	Attitude toward Technology
BI	Behavioral Intention
BI_mean	Composite score of Behavioral Intention
CDSS	Clinical Decision Support System
CFA	Confirmatory Factor Analysis
CS	Clinical Satisfaction
DC	Diagnostic Confidence
DWLS	Diagonally Weighted Least Squares
EE	Effort Expectancy
ED	Emergency Department
FC	Facilitating Conditions
GDPR	General Data Protection Regulation
MDR	Medical Device Regulation
PE	Performance Expectancy
RMSEA	Root Mean Square Error of Approximation
SD	Standard Deviation
SEM	Structural Equation Modeling
SE	Standard Error
SI	Social Influence
SRMR	Standardized Root Mean Square Residual
TLI	Tucker–Lewis Index
UTAUT	Unified Theory of Acceptance and Use of Technology

## Appendix A. Additional Tables

This appendix collates the full numeric outputs and supplementary visualizations referenced in the main text. Raw R scripts and CSV files are available from the corresponding author upon reasonable request.

**Table A1:** Regression effects of gender on latent variables and BI.

Dependent	Estimate	SE	Z	*p*
PE	0.065	0.247	0.265	0.791
EE	0.420	0.274	1.535	0.125
FC	0.041	0.241	0.172	0.864
SI	0.522	0.328	1.589	0.112
AT	0.430	0.284	1.512	0.131
WE	0.258	0.277	0.931	0.352
BI	0.395	0.323	1.221	0.222
DC	0.431	0.269	1.605	0.109
CS	0.476	0.268	1.780	0.075
BI_mean	−0.085	0.229	−0.369	0.712

**Table A2:** Regression effects of clinical experience on latent variables and BI.

Dependent	Estimate	SE	Z	*p*
PE	−0.455	0.250	−1.822	0.069
EE	−0.052	0.306	−0.169	0.866
FC	0.497	0.240	2.075	0.038
SI	0.119	0.293	0.407	0.684
AT	0.174	0.292	0.595	0.552
WE	−0.552	0.253	−2.176	0.030
BI	−0.289	0.293	−0.985	0.325
DC	−0.561	0.247	−2.269	0.023
CS	−0.296	0.267	−1.110	0.267
BI_mean	−0.064	0.218	−0.292	0.770

**Table A3:** Regression effects of experience with AI-based fracture detection on latent variables and BI.

Dependent	Estimate	SE	Z	*p*
PE	0.385	0.271	1.419	0.156
EE	0.777	0.291	2.671	0.008
FC	0.114	0.243	0.472	0.637
SI	0.182	0.300	0.609	0.543
AT	0.312	0.283	1.099	0.272
WE	0.255	0.241	1.056	0.291
BI	0.406	0.294	1.381	0.167
DC	0.217	0.258	0.842	0.400
CS	0.142	0.252	0.562	0.574
BI_mean	−0.233	0.244	−0.955	0.340

**Table A4:** Comparative standardized path coefficients (β) for PE, EE, FC, and SI across selected AI-UTAUT studies.

Study	Setting	PE	EE	FC	SI	BI	Main Findings
This study	ED	0.59	0.20	0.26	0.39	—	PE strongest predictor
Ratta et al. (2025) [30]	Tertiary hospital	0.47	0.22	0.18	0.35	—	SI significant
Cheng et al. (2022) [31]	Dentistry	0.51	0.19	0.24	0.12	—	SI mediated via trust
Wang et al. (2020) [33]	Wearables	0.44	0.31	0.40	0.28	—	FC critical for adoption
Fujimori et al. (2022) [11]	ED	0.61	0.25	0.30	0.18	—	PE and EE dominate
Dingel et al. (2024) [35]	Mixed-clinic	0.55	0.15	0.22	0.28	—	Meta-analysis concordant
Lee et al. (2025) [37]	Acute care	0.50	0.18	0.27	0.33	—	SI resurfaces in teamwork

## References

[1] Kim Y.J., Choi J.H., Fotso G.M.N. (2024). Medical professionals’ adoption of AI-based medical devices: UTAUT model with trust mediation. J. Open Innov. Technol. Mark. Complex..

[2] Pesapane F., Hauglid M.K., Fumagalli M., Petersson L., Parkar A.P., Cassano E., Horgan D. (2025). The translation of in-house imaging AI research into a medical device ensuring ethical and regulatory integrity. Eur. J. Radiol..

[3] Najjar R. (2023). Redefining radiology: A review of artificial intelligence integration in medical imaging. Diagnostics.

[4] Tran A.Q., Nguyen L.H., Nguyen H.S.A., Nguyen C.T., Vu L.G., Zhang M., Vu T.M.T., Nguyen S.H., Tran B.X., Latkin C.A. (2021). Determinants of intention to use artificial intelligence-based diagnosis support system among prospective physicians. Front. Public Health.

[5] Ting D.S.W., Pasquale L.R., Peng L., Campbell J.P., Lee A.Y., Raman R., Tan G.S.W., Schmetterer L., Keane P.A., Wong T.Y. (2019). Artificial intelligence and deep learning in ophthalmology. Br. J. Ophthalmol..

[6] Shan T., Tay F., Gu L. (2021). Application of artificial intelligence in dentistry. J. Dent. Res..

[7] Bhatnagar A., Kekatpure A.L., Velagala V.R., Kekatpure A. (2024). A Review on the Use of Artificial Intelligence in Fracture Detection. Cureus.

[8] Niazi M.K.K., Parwani A.V., Gurcan M.N. (2019). Digital pathology and artificial intelligence. Lancet Oncol..

[9] Vearrier L., Derse A.R., Basford J.B., Larkin G.L., Moskop J.C. (2022). Artificial Intelligence in Emergency Medicine: Benefits, Risks, and Recommendations. J. Emerg. Med..

[10] Zech J.R., Santomartino S.M., Yi P.H. (2022). Artificial Intelligence (AI) for Fracture Diagnosis: An Overview of Current Products and Considerations for Clinical Adoption, From the AJR Special Series on AI Applications. AJR Am. J. Roentgenol..

[11] Fujimori R., Liu K., Soeno S., Naraba H., Ogura K., Hara K., Sonoo T., Ogura T., Nakamura K., Goto T. (2022). Acceptance, Barriers, and Facilitators to Implementing Artificial Intelligence–Based Decision Support Systems in Emergency Departments: Quantitative and Qualitative Evaluation. JMIR Form. Res..

[12] Fan W., Liu J., Zhu S., Pardalos P.M. (2020). Investigating the impacting factors for the healthcare professionals to adopt artificial intelligence-based medical diagnosis support system (AIMDSS). Ann. Oper. Res..

[13] Longoni C., Bonezzi A., Morewedge C.K. (2019). Resistance to medical artificial intelligence. J. Consum. Res..

[14] Act A.I. (2021). Proposal for a regulation of the European Parliament and the Council laying down harmonised rules on Artificial Intelligence (Artificial Intelligence Act) and amending certain Union legislative acts. EUR-Lex-52021PC0206.

[15] Venkatesh V., Morris M.G., Davis G.B., Davis F.D. (2003). User acceptance of information technology: Toward a unified view. Mis Q..

[16] Ammenwerth E. (2019). Technology acceptance models in health informatics: TAM and UTAUT. Applied Interdisciplinary Theory in Health Informatics.

[17] Lambert S.I., Madi M., Sopka S., Lenes A., Stange H., Buszello C.-P., Stephan A. (2023). An integrative review on the acceptance of artificial intelligence among healthcare professionals in hospitals. NPJ Digit. Med..

[18] Wang J., Li X., Wang P., Liu Q., Deng Z., Wang J. (2021). Research trend of the unified theory of acceptance and use of technology theory: A bibliometric analysis. Sustainability.

[19] Hailu D.T., Melaku M.S., Abebe S.A., Walle A.D., Tilahun K.N., Gashu K.D. (2025). A modified UTAUT model for acceptance to use telemedicine services and its predictors among healthcare professionals at public hospitals in North Shewa Zone of Oromia Regional State, Ethiopia. Front. Digit. Health.

[20] Kelkay J.M., Wubante S.M., Anteneh D.S., Takilo M.K., Gebeyehu C.D., Alameraw T.A., Gashu K.D. (2025). Intention to use eLearning-based continuing professional development and its predictors among healthcare professionals in Amhara region referral hospitals, Ethiopia, 2023: Using modified UTAUT-2 model. BMC Health Serv. Res..

[21] Palas J.U., Sorwar G., Hoque M.R., Sivabalan A. (2022). Factors influencing the elderly’s adoption of mHealth: An empirical study using extended UTAUT2 model. BMC Med. Inform. Decis. Mak..

[22] Zhang Q., Zhang R., Lu X., Zhang X. (2023). What drives the adoption of online health communities? An empirical study from patient-centric perspective. BMC Health Serv. Res..

[23] Chismar W.G., Wiley-Patton S. Does the extended technology acceptance model apply to physicians. Proceedings of the 36th Annual Hawaii International Conference on System Sciences.

[24] Liyanage H., Liaw S.-T., Jonnagaddala J., Schreiber R., Kuziemsky C., Terry A.L., de Lusignan S. (2019). Artificial intelligence in primary health care: Perceptions, issues, and challenges. Yearb. Med. Inform..

[25] Jiang L., Wu Z., Xu X., Zhan Y., Jin X., Wang L., Qiu Y. (2021). Opportunities and challenges of artificial intelligence in the medical field: Current application, emerging problems, and problem-solving strategies. J. Int. Med. Res..

[26] Venkatesh V. (2022). Adoption and use of AI tools: A research agenda grounded in UTAUT. Ann. Oper. Res..

[27] Wu J., Lederer A. (2009). A meta-analysis of the role of environment-based voluntariness in information technology acceptance. Mis Q..

[28] Wolf E.J., Harrington K.M., Clark S.L., Miller M.W. (2013). Sample size requirements for structural equation models: An evaluation of power, bias, and solution propriety. Educ. Psychol. Meas..

[29] Rhemtulla M., Brosseau-Liard P.É., Savalei V. (2012). When can categorical variables be treated as continuous? A comparison of robust continuous and categorical SEM estimation methods under suboptimal conditions. Psychol. Methods.

[30] Ratta R., Sodhi J., Saxana U. (2025). The Relevance of Trust in the Implementation of AI-Driven Clinical Decision Support Systems by Healthcare Professionals: An Extended UTAUT Model. Electron. J. Knowl. Manag..

[31] Cheng M., Li X., Xu J. (2022). Promoting healthcare workers’ adoption intention of artificial-intelligence-assisted diagnosis and treatment: The chain mediation of social influence and human–computer trust. Int. J. Environ. Res. Public Health.

[32] Kumar A., Kumar D.V.S., Sinha P., Megha R. (2024). Customer Acceptance of Artificial Intelligence in Healthcare: A Systematic Literature Review and Proposition of Conceptual Framework for Future Research. Res. Sq..

[33] Wang H., Tao D., Yu N., Qu X. (2020). Understanding consumer acceptance of healthcare wearable devices: An integrated model of UTAUT and TTF. Int. J. Med. Inform..

[34] Su J., Wang Y., Liu H., Zhang Z., Wang Z., Li Z. (2025). Investigating the factors influencing users’ adoption of artificial intelligence health assistants based on an extended UTAUT model. Sci. Rep..

[35] Dingel J., Kleine A.-K., Cecil J., Sigl A.L., Lermer E., Gaube S. (2024). Predictors of Health Care Practitioners’ Intention to Use AI-Enabled Clinical Decision Support Systems: Meta-Analysis Based on the Unified Theory of Acceptance and Use of Technology. J. Med. Internet Res..

[36] Marinescu Ș.A., Oncioiu I., Ghibanu A.-I. (2025). The Digital Transformation of Healthcare Through Intelligent Technologies: A Path Dependence-Augmented–Unified Theory of Acceptance and Use of Technology Model for Clinical Decision Support Systems. Healthcare.

[37] Lee A.T., Ramasamy R.K., Subbarao A. (2025). Understanding Psychosocial Barriers to Healthcare Technology Adoption: A Review of TAM Technology Acceptance Model and Unified Theory of Acceptance and Use of Technology and UTAUT Frameworks. Healthcare.

[38] Tariq A., Purkayastha S., Padmanaban G.P., Krupinski E., Trivedi H., Banerjee I., Gichoya J.W. (2020). Current clinical applications of artificial intelligence in radiology and their best supporting evidence. J. Am. Coll. Radiol..

[39] Bachtiar A. (2024). Maximizing Artificial Intelligence for Patient Satisfaction: Marketing Strategies in The Digital Health Era. Contag. Sci. Period. J. Public Health Coast. Health.

[40] Higgins O., Short B.L., Chalup S.K., Wilson R.L. (2023). Artificial intelligence (AI) and machine learning (ML) based decision support systems in mental health: An integrative review. Int. J. Ment. Health Nurs..

[41] Goddard K., Roudsari A., Wyatt J.C. (2012). Automation bias: A systematic review of frequency, effect mediators, and mitigators. J. Am. Med. Inf. Assoc..

[42] Amann J., Blasimme A., Vayena E., Frey D., Madai V.I., Consortium P.Q. (2020). Explainability for artificial intelligence in healthcare: A multidisciplinary perspective. BMC Med. Inform. Decis. Mak..

